# Global value chains, import orientation, and the state: South Africa’s pharmaceutical industry

**DOI:** 10.1057/s42214-021-00103-y

**Published:** 2021-04-06

**Authors:** Rory Horner

**Affiliations:** 1 Global Development Institute, University of Manchester, Manchester, UK; 2 Department of Geography, Environmental Management and Energy Studies, University of Johannesburg, Johannesburg, South Africa

**Keywords:** global value chains, imports, policy, pharmaceuticals, South Africa

## Abstract

As opposed to the predominant focus of global value chain (GVC) research on export-oriented contexts, this article examines the prospects for development in places where the dominant form of engagement with GVCs is import-oriented. Through the case of South Africa’s pharmaceutical industry, this analysis demonstrates the challenge for local manufacturing to compete, and the associated state policy responses, in a place which is largely plugged into GVCs as an end market rather than as a production location. As multinationals have concentrated production elsewhere, South Africa’s manufacturing capacity in the pharmaceutical industry has relatively declined in recent decades. Having struggled in its facilitator role, the South African state’s efforts to promote local manufacturing have turned to the producer role through a state-owned company and especially the buyer role through public procurement. Motivations for state policy in this context, however, must navigate the tension which sometimes exists between the industrial interest in local manufacturing and the consumer and health policy interest in access to medicines. The experience of South Africa’s pharmaceutical industry points to the wider challenge and consequences of import-oriented engagement with GVCs for local industrial development.

## INTRODUCTION

Contemporary understandings of development with regard to global value chains (GVCs) are largely set within a liberal global economy where export orientation has been the dominant development strategy. To understand local economic development, research on GVCs in development studies, economic geography, economic sociology, and international business has provided meso-level studies of how firms, farmers, and workers are differentially ‘plugged’ into globally organized industries and their prospects for upgrading (e.g., Barrientos, Gereffi, & Rossi, 2011; Gereffi, 1994, 1999; Giuliani, Pietrobelli, & Rabellotti, 2005). In this research, the dominant understanding of the role of the state in GVCs (and global production networks) has been implicitly limited to playing a facilitator role – assisting local firms to participate in GVCs or attracting the activities of global lead firms and their suppliers (Gereffi, 1994; Horner, 2017; Horner & Alford, 2019). Similarly, international business literature has focused on international business promotion and foreign business attraction (Lundan, 2018). Collectively, such approaches tend to assume the pre-existence of, or potential for development of, localized assets that can be exploited for export-oriented development within the global economy (e.g., Coe, Hess, Yeung, Dicken, & Henderson, 2004). Yet, what are the prospects for development and associated state policies in places that are not major centers of manufacturing for global lead firms and that are mainly importers of products produced via GVCs?

Through the case of South Africa’s pharmaceutical industry, where there are relatively few localized assets that can be leveraged for export competitiveness, this article examines the development challenges of an engagement with GVCs which is oriented towards the import of products for domestic consumption. The South African case is fitting because the country’s pharmaceutical manufacturing capacity has been gradually eroded from the 1990s liberalization onwards, with the consequent rise of imports of finished drugs produced elsewhere. Motivated by an industrial interest, the state has struggled to promote local pharmaceutical manufacturing. Although sometimes in tension with a public health motivation, the producer (via state ownership) and buyer (via public procurement) roles have been turned to in order to promote better development outcomes in terms of local industrial development and public health.

The article contributes to research on GVCs by demonstrating the competitive challenge to, and difficulty for state policies to promote, local industrial development in a place that is primarily incorporated into GVCs as an end market, rather than as a production location. This dynamic is not limited geographically, to South Africa, or sectorally, to pharmaceuticals. The formation of GVCs has led to considerable consolidation of production in particular parts of the world in various industries (Gereffi, 2014), changing the prospects for economic development elsewhere, with deindustrialization a trend in many places in recent decades (Pike, 2020; Rodrik, 2016). Yet, despite a flurry of work in both global North (e.g., Autor, Dorn, & Hanson, 2013) and global South (e.g., Jenkins, 2018; Moreira, 2007) on import competition and its implications for economic development, this dynamic has rarely been considered in relation to GVCs. However, situating this challenge of developing local manufacturing in a GVC context provides a deeper understanding of the scale of the challenge facing local producers. Moreover, and building on recent work on the state–GVC nexus (e.g., Alford & Phillips, 2018; Hauge, 2020; Mayer & Phillips, 2017; Smith, 2015; Werner, 2020; Yeung, 2016) and on international business policy (Lundan, 2018; Van Assche, 2018), the article demonstrates the limitations of the state facilitator role in the context of import-oriented engagement with GVCs and the consequent turn to, and also challenges regarding, the producer (state ownership) and buyer (public procurement) roles of the state. Rather than the commonly used term import substitution (e.g., Irwin, 2021), import orientation is referred to here to describe a relationship between territories and GVCs, which involves a range of policies, processes, and strategies, which extend beyond restricting imports of manufactured goods and include encouragement of imported products.

The article builds on recent research on local pharmaceutical production, an issue which has attracted growing policy attention in recent years (e.g., Dong & Mirza, 2016) given a considerable dependence on imported medicines in many countries as a result of pharmaceutical globalization. While COVID-19 has energized policy discussions of promoting greater domestic production of pharmaceuticals in the United States and Europe (Barbieri, Elia, Fratocchi, Kalchschmidt, & Samson, 2020; Hahn & Shah, 2020), a more established body of research has examined the challenge for local production in the global South (e.g., Kaplan & Laing, 2005; Shadlen, 2009; WHO, 2011), especially in sub-Saharan Africa (Chaudhuri, Mackintosh, & Mujinja, 2010; Chorev, 2019; Mackintosh, Banda, Tibandebage, & Wamae, 2015; Vugigi, Thoithi, Ogaji, & Onuonga, 2017). Yet, with only a few exceptions (e.g., Chaudhuri et al., 2010; Haakonsson, 2009), such work has not provided a detailed understanding of the nature of import competition faced or of how local producers relate to GVCs. Here, the dynamics of GVCs are demonstrated to be crucial to understanding the magnitude of the competitive challenge that local producers face.

The article proceeds as follows. Following a review of the export-oriented focus and state facilitator role of much research on GVCs and development, the next section considers the alternative dynamic concerning import-oriented engagement with GVCs. The research methods are then outlined, including primary interviews with senior representatives of key industry stakeholders, and the research context is introduced. The case study then demonstrates the trajectory of South Africa’s pharmaceutical industry from some manufacturing capacity pre-liberalization, to relative manufacturing decline and increasing import reliance. State responses are then outlined, from the struggle with the facilitator role to the producer and buyer role, before the article concludes.

## GVCS, STATES, AND DEVELOPMENT: BEYOND EXPORT-ORIENTED DEVELOPMENT AND THE FACILITATOR ROLE

Dating back to the Washington Consensus emphasis on privatization, stabilization, and liberalization from the late 1980s onwards, export orientation has been the dominant economic development strategy across most of the world. In the context of this liberalized global economy, the dominant form of industrial organization is GVCs, where the productions of goods and services has become fragmented amongst different actors as well as across countries and continents.

From an interest in economic development, predominantly in the global South, an increasingly vast body of research has examined the varying possibilities of export-oriented development for firms, farmers, and territories within GVCs. Originating with work on what were then termed global commodity chains (GCCs), and evolving to GVCs, such research has sought to understand the prospects for economic (e.g., Gereffi, 1994, 1999), social (e.g., Barrientos et al., 2011) and/or environmental (e.g., De Marchi, Di Maria, Krishnan, & Ponte, 2019) upgrading of suppliers within such chains. A key question has been how and under what conditions upgrading occurs, with an emphasis on the influence of lead firms and how they control or govern their chains (e.g., Buckley & Strange, 2015; Gereffi, Humphrey, & Sturgeon, 2005; Giuliani et al., 2005; Hernández & Pedersen, 2017; Humphrey & Schmitz, 2002). Closely related research on global production networks (GPNs) has also examined possibilities for export-oriented development through “strategic coupling” between localized assets and the needs of lead firms in GPNs (Coe & Yeung, 2015; Coe et al., 2004; Yeung, 2016). Research on both GVCs and GPNs has also demonstrated the struggle for many producers and places to achieve export-oriented upgrading (Blažek, 2016; Gibbon & Ponte, 2005; Horner, 2014; Neilson & Pritchard, 2009; Pipkin & Fuentes, 2017).

With an emphasis on breaking with state-centric approaches to understanding economic development by focusing on private governance (Mayer & Phillips, 2017), the role of the state has received less explicit attention in research on GVCs, at least until recently. The flourishing of research on GVCs occurred in a context of a switch from import-substitution to export-orientation as a dominant development strategy (Bair, 2005: 161), with the role of the state mostly understood as a facilitator (Gereffi 1994: 100; Horner, 2017). Although the original GPN conceptual framework sought to give greater explicit attention to the role of non-firm actors, including the state (Coe et al., 2004; Henderson, Dicken, Coe, Hess, & Yeung, 2002), self-reflection by its proponents has acknowledged that such literature has also overlooked the state to some extent (Coe, Dicken, & Hess, 2008; also Smith, 2015). Even the international business literature, which had taken an earlier interest in MNE–state relationships, also took a firm-centric approach and largely overlooked questions of public policy (Gereffi, 2019; Van Assche, 2018). The goals of states in this export-oriented context were either to facilitate local businesses to embed in a multinational enterprise’s (MNE) network or to attract MNEs (Dicken, 1994: 123) and were encouraged further by the shrinking of “development space” arising from the restrictions on the use of various policies by the World Trade Organization (WTO) (Wade, 2003).

A growing body of more recent work, however, has begun to focus on a range of state roles in GVCs beyond that of facilitator, including as regulator, producer, and buyer (Horner & Alford, 2019). For example, the regulator role has received attention in terms of the interactions between public and private governance (e.g., Bartley, 2018), as well as regarding the influence of tariffs (Curran, Nadvi, & Campling, 2019). The role of the state as a producer through state-owned companies, although a greater emphasis in international business research (e.g., Cuervo-Cazurra, Inkpen, Musacchio, & Ramaswamy, 2014), has still to receive considerable attention in relation to GVCs. Nevertheless, emerging research has focused on the state buyer role in terms of public procurement (Hughes, Morrison, & Ruwanpura, 2019). More broadly, growing attention has been placed on the active ways states are shaping their engagement with global trade (Evenett, 2019), including through industrial policy (Hauge, 2020; Kaplinsky & Morris, 2015; Whitfield, Staritz, & Morris, 2020).

The consequences of the importation of goods, produced through GVCs, for local industrial development in the end markets they are supplied to has largely been overlooked in GVC research. Such work has mostly focused on the development implications for firms which are already part of these chains, and has consequently been critiqued for neglecting the development processes and challenges that occur outside, yet are still crucially related to, such chains as part of an “inclusionary bias” (Bair & Werner, 2011). The next section fosters an understanding of the development implications of an import-oriented engagement with GVCs.

## IMPORT ORIENTATION AND GLOBAL VALUE CHAINS

Given the above export-oriented focus, research on GVCs has overwhelmingly considered territories in the global South as sites of production rather than as end markets for the sale of products produced via GVCs. A body of research in international economics on trade-in-value added has analyzed the role of imports of intermediate goods (e.g., Baldwin, 2016), but mostly in terms of subsequent export and without the attention to sector-specific relationships, actors, and processes. Although a more recent body of research in development studies and economic geography has begun to consider Southern end markets for finished products (Haugen, 2018; Horner & Murphy, 2018; Kaplinsky, Terheggen, & Tijaja, 2011; Pasquali, 2020; Staritz, Gereffi, & Cattaneo, 2011), that is still primarily from the view of producers seeking to export. Nevertheless, one study has made a useful distinction between outward coupling related to exports and inward coupling related to the flow of imports for local consumption and which shapes local industrial regimes. In the wood products industry in Tanzania and South Africa, imports have created challenges for local firms to compete in a relatively liberal trade context (Murphy & Carmody, 2015).

Separate to the literature on GVCs, a body of work has documented some of the development implications of import competition. A significant de-industrialization trend in the era of globalization has been found not just in the global North (e.g., Autor et al., 2013; Krugman, 2008), but also in the global South. Africa and Latin America have been particularly affected, and trade has been prominently pointed to as a cause of such deindustrialization (Rodrik, 2016). Manufacturing industries largely serving domestic end markets, often previously established and existing in a context of segmentation from global markets by higher transport costs and trade and industrial policies oriented towards import-substitution, face a competitive threat. The rise of Asian economies, especially China and to a lesser extent India, have posed a significant challenge for new and existing industries elsewhere, and especially in the global South (Jenkins & Edwards, 2015; Kaplinsky & Messner, 2008).

Considering a GVC lens together with prior work on import competition deepens understanding of development challenges in an import-oriented context. Research on import competition and deindustrialization in the global South has focused on the examination of trade data and the manufacturing sector as a whole, rather than sector case studies or the examination of different stages of activity involved in production and distribution as befits a value chain analysis. By considering the industrial organization of GVCs, research on (de-)industrialization can draw more refined, sector-specific understandings of the possibilities for economic development and associated state strategies. Such work can also help identify particular segments of an industry which may be more opportune (or not) to enter and the associated barriers to do so, as well as better inform the scale of the challenge to be met in order to be competitive.

The rise of GVCs in a context of trade liberalization has involved consolidation and concentration of global production (Gereffi, 2014), leading many territories to rely on the importation of finished products. Lead firms benefit from economies of scale through such concentration and seek a wide range of end markets for their products. In a liberalized trade context with few barriers to imports, local producers (whether domestically or foreign-owned) seeking to supply a given domestic market must then compete with imported products, which may have been produced with the economies of scale that characterize global lead firms producing through GVCs.

Policymakers seeking to respond to this situation must devise strategies attuned to different stages of an industry (Whittaker, Sturgeon, Okita, & Zhu, 2020) and going beyond the facilitator role. This response must also deal with potential tensions between consumer and producer interests (Altenburg 2006: 510). Although often overlooked in research on GVCs, consumers may benefit from imported products if they are cheaper, better quality, or offering products hitherto unavailable (Kaplinsky & Messner, 2008: 204). At the same time, a longer-term perspective on producer interests may also consider factors such as learning opportunities and longer-term prospects for structural change, which can initially arise through participating in domestic and regional markets (Altenburg, 2006: 510; Hauge, 2020). Imports of intermediate goods can be used as inputs and may complement local manufacturing to some degree (Edwards & Jenkins, 2015). Thus, state motivations beyond capital accumulation, and the possibilities for struggle within the state (Smith, 2015), must be recognized as part of how states approach international business (Van Assche, 2018), GVCs, and development.

Building on these insights, the analysis of the case of South Africa’s pharmaceutical industry departs from an export-oriented context with the state playing a facilitator role, to instead examine development in a context of a predominantly import-oriented engagement with GVCs. The case study involves the state moving beyond both its role as a facilitator to the buyer and producer roles, and from a focus on encouraging formulation production to move upstream in the value chain towards active pharmaceutical ingredient production.

## CASE AND METHODOLOGY

The analysis here focuses on the South African pharmaceutical industry, the largest such industry in sub-Saharan Africa in revenue, although relatively small in global terms. Total pharmaceutical sales in South Africa were estimated at US $3.428 billion in 2018 (BMI Research, 2019: 5). Generic drug sales were estimated at US $1.251 billion in 2018, and their share has been forecast to grow over the long term with single-exit pricing, a growing middle class, and an emphasis on cost containment (BMI Research, 2019: 19). The biggest sellers in monetary (as opposed to volume) terms are treatments for hypertension and heart disease, even though HIV/AIDS is the biggest killer (BMI Research, 2019: 34). With a bifurcated health system as a legacy of apartheid (Government of South Africa, 1996: 3), the public sector is a very significant part of the pharmaceutical market in South Africa, estimated at 31% of the just over US $3 billion industry in 2017 (Helen Suzman Foundation, 2018: 3). Nevertheless, some estimates have put the public sector demand at up to 75% in volume terms, which more than 80% of the population relies on (Kudlinski, 2013: 268). Table 1 below outlines the top firms by products registered in 2017.

**Table 1:** Top five firms by number of products registered in the South African private and public pharmaceutical market, 2017 Source: Adapted from Helen Suzman Foundation (2018: 3). Number of products in brackets

Private	Public
Aspen Pharmacare (601)	Aspen Pharmacare (106)
Adcock Ingram (401)	Fresenius Kabi (53)
Sandoz (338)	Cipla Medpro (58)
Cipla Medpro (223)	Sanofi Aventis (55)
Pfizer (219)	Adcock Ingram Critical Care (52)

Like all countries, South Africa has both health and industrial interests in pharmaceuticals. The former is largely the preserve of the Department of Health (DoH), the latter of the Department of Trade and Industry (DTI). The 1996 National Drug Policy, which stated health, economic, and national development objectives, has been influential in shaping the policy approach to pharmaceuticals in post-apartheid South Africa. The key health objectives included availability, accessibility, and quality of drugs. One of the national development objectives included “to support the development of the local pharmaceutical industry and the local production of essential drugs” (Government of South Africa 1996: 4). While GVC research has largely focused on the implications for producers from integration into GPNs, serving local consumers (i.e., patients) weighs heavily in shaping the policy approaches to pharmaceuticals in South Africa and is sometimes in tension with the industrial interest in local manufacturing.

The analysis draws on primary research conducted from July to November 2014, as well as in February to March 2019, totaling 30 elite interviews with pharmaceuticals stakeholders in South Africa (Greater Johannesburg-Pretoria, Cape Town and Durban), as well as attendance and conversations at an industry exhibition in Sandton. Table 2 provides background information on the interviewees. The firm-level interviews were with senior representatives of, mostly South African and Indian-owned, firms present in the generics segment of the industry – key for volumes and for local manufacturing. Potential interviewees were identified from a list of members of the National Association of Pharmaceutical Manufacturers (since 2017, Generic and Biosimilar Medicine SA), as well as from a reading of secondary literature. The interviews focused on challenges and opportunities for the generics-focused local pharmaceutical manufacturing in South Africa, as well as the involvement and influence of Indian pharmaceuticals as the major source of imports. The interviews were audio-recorded where possible (19 of the 30), with hand-written notes taken where this was not possible. Interview materials were transcribed and then coded using NVivo9 qualitative data analysis software according to entry barriers, challenges for local manufacturing, value chain partnerships, exports, access to medicines, as well as the roles of the state (facilitator, regulator, producer, buyer). Secondary literature, including government policy reports, corporate websites, media, and other academic literature was consulted to validate and triangulate the material gathered through interviews, and provided more historical depth and coverage of the involvement of research-based MNEs (who were difficult to access through interviews). Given there are few publicly available comprehensive data sources on pharmaceuticals in South Africa, the case that follows draws on an extensive range of sources from the secondary literature.

**Table 2:** Background information on the stakeholders interviewed

Firms in South Africa’s pharmaceutical market (16)	
Ownership	South Africa: 8
	India (> 50% stake): 7
	Elsewhere: 1
Local manufacturing	Yes: 8
	No: 8
Rank of person interviewed	Country Manager: 4
	Chief Executive Officer: 4
	Managing Director: 2
	Chief Financial Officer: 1
	Chief Operation Officer: 1
	Senior Pharmacist: 1
	Senior Trade Executive: 1
	Exports & Tenders Manager: 1
	Business Development Manager 1
Other interviewees (14)	
	Consulting companies: 3
	Industry association groups: 1
	Policy organizations: 3
	Civil society organizations: 2 Aid-funded programme: 1 Academic experts: 3 Pharmaceutical journalist: 1

**Figure 1:**
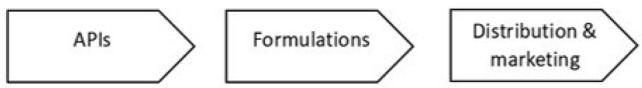
A simplified pharmaceutical value chain. Source: Author’s construction.

In terms of industry context, a simplified illustration of the main stages of a typical pharmaceutical value chain is provided in Figure 1 below. In terms of the manufacturing stage of the value chain, a key distinction can be made between the production of active pharmaceutical ingredients (APIs) and finished drug formulations. API production is a chemical process involving reactors for drug substance manufacture and has high entry barriers. The second main stage is formulations production, which is a physical process of adding excipients to an API and ‘formulating’ the drug into a consumable form such as a tablet, liquid, capsule, cream, ointment, or injectable. The entry barriers are much lower for this stage. Distribution and marketing follow the completion of manufacturing. As the South African case below illustrates, the country’s pharmaceutical manufacturing is almost entirely focused on the formulation stage of the value chain. This means almost all APIs still need to be accessed from abroad for the local manufacturing which does take place within the country, which also must compete with finished drugs produced elsewhere in places which likely have privileged access to APIs and other raw materials.

## FROM LOCAL PHARMACEUTICAL MANUFACTURING TO IMPORT ORIENTATION IN SOUTH AFRICA’S PHARMACEUTICAL INDUSTRY

In this section, the development of the South African pharmaceutical industry is discussed in three sub-sections. The first is the period of localized manufacturing before liberalization, the second involves some deindustrialization under liberalization as global pharmaceutical production has been consolidated elsewhere, and, finally, the third documents the growing orientation towards imports of finished products. The various state responses to deindustrialization and import growth in terms of how to promote ‘better’ development outcomes are then outlined in the following section.

### Local Pharmaceutical Formulation Manufacturing Pre-liberalization

The modern pharmaceutical industry in South Africa grew from retail pharmacists who initially dominated the industry in the late 19th and early 20th century. Appointed as local distribution agents for foreign firms, they mostly imported finished products from abroad, although some also ventured into very basic manufacturing. For example, what is now South Africa’s largest pharmaceutical company, Aspen Pharmacare, traces its roots to Lennon Ltd., a pharmacy established in Port Elizabeth in 1850. The second largest South African pharmaceutical firm today, Adcock Ingram, traces its origins to the E.J. Adcock Pharmacy, which opened in Krugersdorp in 1890.

Local pharmaceutical formulation manufacturing expanded from the mid-20th century in South Africa in a context where pharmaceutical MNEs, there and elsewhere, often established manufacturing plants alongside marketing, sales, and distribution functions. World War II led to an increased demand for medicines and expansion of local manufacturing in the country (Ryan, 1983: 87). For example, Adcock Ingram opened its first manufacturing facility at Aeroton in 1940 and increased its scale with a licensing agreement from Baxter in 1948. By the end of the 1950s, 70% of medicines were imported and there were 32 subsidiaries of foreign MNEs and 33 foreign companies locally represented by agencies or distributors (Davenport-Hines & Slinn, 1992: 317). Examples of MNEs that opened plants include Glaxo in 1954 at Wadeville Germiston (Davenport-Hines & Slinn, 1992: 319–320), Pfizer in 1968 at Pietermaritzburg (Pfizer South Africa, 2020), and what is now Sanofi-Aventis in 1972 at Pretoria (Sanofi South Africa, 2020). In an example of locally-owned manufacturing, South African Druggists took over Lennon Ltd. and, on a site in Port Elizabeth where it had been in operation since approximately 1940, significantly upgraded its manufacturing and research facilities between 1975 and 1988 (Aspen, 2005: 34). One estimate is that by the 1980s, approximately 45 pharmaceutical manufacturing facilities were present in South Africa (Focus Reports, 2012: 15).

The manufacturing aspect of the South African pharmaceutical industry has historically overwhelmingly focused on the formulations stage of the value chain, assembling final products from imported active and inactive ingredients (Steenkamp 1979). In a limited example of a move into active pharmaceutical ingredients (API) production, Fine Chemicals was established in Cape Town in 1962, initially targeting the production of codeine phosphate, morphine sulphate, and paracetamol (Fine Chemicals Corporation website, accessed March 3, 2020). Yet, with only 13% of the APIs used in South Africa in the late 1970s being produced locally, and those too requiring imported fine chemicals, there was a considerable overall dependence on imports – even for products where the formulation stage was local (Steenkamp, 1979: 86).

Foreign-owned pharmaceutical MNEs from the United States and Europe dominated the South African pharmaceutical market through most of the second half of the 20th century, benefiting from the huge technological progress (e.g., antibiotics revolution) in the global pharmaceutical industry between 1940 and 1955 (Chandler, 2005; Gereffi, 1983). Of the market for prescription medicines, by the late 1970s, foreign-owned firms had an 85.8% share of the market, led by those from the US (39.2%), UK (18.2%), Switzerland (14.4%), and Germany (7.5%) (Steenkamp, 1979: 76). Multinationals faced lobbying pressure from the anti-apartheid movement to leave South Africa, and although Merck did leave during 1989, many others including Eli Lilly, Johnson & Johnson, Pfizer, Schering-Plough, and Warner Lambert, continued to operate in the country (Prakash Sethi & Williams, 2012).

### Multinational-led Deindustrialization and the Emergence of Import-oriented Engagement with GVCs

Since the opening up of the South African economy post-apartheid, the country’s manufacturing industry has struggled to compete, including in pharmaceuticals. Manufacturing value added as a share of GDP, steady at between 19 and 22% of GDP from 1960 to 1990, has fallen from 21.6% in 1990 to 17.5% in 2000 to 12.1% in 2010 and 11.8% by 2018 (World Bank World Development Indicators, accessed March 2, 2020). Within pharmaceuticals, some European and US-owned MNEs have ‘functionally downgraded’ their South African operations out of manufacturing. In the first decade post-apartheid, it is widely estimated that more than 30 pharmaceutical manufacturing plants closed, with the direct loss of 6500 jobs (e.g., Maloney & Segal, 2007: 6). Table 3 below provides examples of some of the closures of pharmaceutical plants in South Africa in the late 1990s. Other companies not listed in Table 3 which closed manufacturing plants in South Africa in the years leading up to and following the turn of the Millennium included AstraZeneca, Novo Nordisk, Schering-Plough, Eli Lilly, Roche, Abbot, and GlaxoSmithKline (Johannesburg factory only) (Maloney & Segal, 2007: 31).

**Table 3:** Closures of domestic pharmaceutical plants in South Africa in the late 1990s Source: Adapted from Financial Mail, 31 July 1998 (contained in Fridge, 2000: 46)

Company	Location	Jobs lost	Reason
Searle	Johannesburg	77	Restructuring post Monsanto merger
Pharmacia/Upjohn	Isando	75	Merger between the companies
Bristol Myers Squibb	Wadeville	50	Merger between the companies
Wellcome	Spartan	150	Restructuring-merger with Glaxo
Adcock Ingram	Various	1000	Merger with Prempharm
Boots	Isando	Unknown	Company bought out by Knoll
Noristan	Pretoria	Unknown	Company bought out by Hoechst
Wyeth	Isando	Unknown	Internal restructuring

Many pharmaceutical MNEs withdrew from manufacturing in South Africa in order to concentrate production at “centers of excellence” elsewhere, involving large, lower-cost units benefiting from economies of scale and serving global markets (Fridge, 2000: 138; various interviews). Domestic pharmaceutical plants were built in an era of sanctions and involved outdated technology, as well as limited volume capacity (Fridge, 2000: 55). One report found that many respondents from pharmaceutical MNEs believed it would be more efficient to limit manufacturing activities in South Africa to packaging and labeling (Fridge, 2000: 84), while others exited from any manufacturing activity in the country. The small size of the South African market meant that it had little importance in size terms for many MNEs (CEO of Novartis SA, in Focus Reports, 2006: S27). The General Manager of Bayer Healthcare, which shifted to produce all of its over-the-counter and pharmaceutical products for South Africa from Germany, was quoted as saying that companies had scaled down production because “it has become more efficient to import drugs” (Focus Reports, 2006: S27). Deloitte (2007: 21) referred to “the closure of manufacturing plants in the country…. due to the global consolidation of supply chains and centers of excellence”. The concentration of production was augmented by considerable mergers and acquisitions in the global pharmaceutical industry (Maloney & Segal, 2007: 31; DTI 2011: 20; also DTI, 2014: 94). As well as their presence declining, foreign MNEs' reputation was also especially damaged by a high-profile and controversial legal challenge (launched in 1998 and eventually abandoned in 2001) to reforms to South Africa’s Medicines and Related Substances Control Act, which would facilitate generic entry (Horner, 2015).

Although many European and US-owned MNEs consolidated their manufacturing elsewhere, some maintained limited manufacturing presence in South Africa for niche production lines. For example, Roche found South Africa “too small to qualify” as a center of excellence (Deloitte, 2007: 38), but decided to manufacture Fansidar in South Africa. Sanofi-Aventis invested R20 million in an expansion of its anti-TB drugs facility in Waltloo/Mamelodi (formerly Noristan Pharmaceuticals) in 2010 (DTI, 2011: 22), while GSK upgraded its Albendazole plant in Cape Town (DTI, 2011: 21).

Table 4 below outlines 26 companies with local manufacturing in South Africa, involving a total of 32 plants, in 2011. Such manufacturing plants varied in employment from 20 (Bioclones and Gulf Drug Co.) to 2500 (Aspen-Pharmacare in Port Elizabeth) (DTI, 2011: 19). This is a considerably smaller number of plants than had been present during the apartheid era, and many of those plants which remain are widely estimated to operate well below full capacity (e.g., Focus Reports, 2006: S27; Pharasi et al., 2010: 80; Interview, Cape Town, February 27, 2019). A 2018 estimate for the number of direct jobs in the manufacturing segment of the industry is 9600 (DTI, 2018: 141).

**Table 4:** Pharmaceutical manufacturing in South Africa, 2011

Activity	Firms present
Manufacture of APIs and intermediates	Fine Chemicals Corp.; Sanofi-Aventis (for captive market); Shimoda, Bioclones (both biological active ingredients); National Bioproducts Institute (plasma-derived medicines)
Parenterals	Adcock-Ingram Critical Care; Fresenius Kabi (2); Dismed Criticare; Thusanong Health Care
Medical textiles	BBSN Medical
Formulated pharmaceuticals	Adcock-Ingram (2); Aspen-Pharmacare (2); Johnson and Johnson (2); Cipla-Medpro; GSK; Gulf; NBI; Pharma Dynamics; Pharma Q; Portfolio; Ranbaxy Be-Tabs; Roche; Sanofi-Aventis; Specpharm
Packaging and labeling (e.g., importing of tablets, capsules, and putting in packs, bottles)	Merck, Sharp and Dohme; Biotech Pharma; Phambili-Dismed

Source: Adapted from DTI (2011: 19–20)

Some locally owned firms moved into the market space, and sometimes physical plants, where foreign MNEs had been operating and have become the largest firms in the South African pharmaceutical market through acquisition. Adcock Ingram acquired parts or all of Baxter, Dow-Chemicals Africa, Restan Laboratories, Stirling Winthrop (all in the 1980s), Leppin, Laser, Pharmatec, Zurich Pharmaceuticals, Covan Pharmaceuticals and Salters (in the 1990s) and Parke-Med (Pfizer’s generic business in 2003). Aspen took over South African Druggists in 1999 (which had a manufacturing site at Port Elizabeth), a company which in turn had acquired Lennon Ltd. in the 1970s, and the API-producer Fine Chemicals in 2004. Local contract manufacturers, such as Wrapsa founded in 1983 and PharmaQ similarly in 1998, have also filled some of the void left by companies who have ceased to manufacture in their own name in South Africa.

The almost complete absence of manufacturing presence in the API stage of the value chain in South Africa, owing to constraints of scale and cost, has been a constraint on the expansion of formulations production. The lack of API production was referred to as “the ‘Achilles heel’ of South Africa’s pharmaceutical industry” (Kudlinski, 2011). Local API production has always been limited in South Africa, with the Cape Town-based, Aspen subsidiary, Fine Chemicals the main manufacturing presence in South Africa in this segment of the value chain. One estimate from 2011 was that at least 95% of the APIs were sourced through imports (DTI, 2011: 19). Lack of greater local API production in South Africa has been attributed to “fierce competition from low-cost Indian and Chinese imports” (DTI, 2014: 95). In addition, the lack of economies of scale in South Africa is a major constraint (Fridge, 2000: 57). API production is specialized according to different drugs, with many API factories needed for a portfolio of drugs (Maloney & Segal, 2007: 63). One interviewee from a contract manufacturing company explained that “the market is too small for APIs in South Africa”. Illustrating with an example, the interviewee stated that “You can buy paracetamol way cheaper elsewhere. You have to produce millions of tons of the stuff. You will never in South Africa” (Interview, Centurion, October 27, 2014).

Due to the reliance on imports for APIs and other imported inputs, even drugs where the final formulation manufacturing takes place in South Africa involve considerable foreign value-added (Maloney & Segal, 2007: 96). One estimate was that typical costs for a product manufactured in South Africa were 40% domestic (e.g., labor, utilities, overhead) and 60% international (90% of that being APIs, the rest being other imported inputs, e.g., packaging materials), although in some cases (e.g., anti-retrovirals (ARVs)) the international costs could rise to 80–90% (Maloney & Segal, 2007: 94). Another estimate of the extent of local content suggested it varies between 10 and 20% where only packing activity takes place locally, 40–70% for finished formulation based on imported API (40 and 70%), and 90%+ for large-volume parenterals (DTI, 2011: 24). With the reliance on imports, including of inputs, the industry has also suffered from exchange rate depreciation (e.g., Focus Reports, 2012: 12). Domestic producers face tariffs on APIs, while a producer outside South Africa may not and may be able to import a formulation tariff-free (Maloney & Segal, 2007: 96). With such an extent of imported inputs, some interviewees thus questioned how ‘local’ local manufacturing really is (e.g., Interviews, Midrand, July 16, 2014; Sandton, October 31, 2014).

### The Consolidation of Import-orientation with Indian Generics

With the liberalization of the economy, South Africa has struggled to export pharmaceuticals and the reliance on imported products, which can enter tariff-free (DTI, 2018: 142), has grown. South Africa’s trade deficit in pharmaceuticals (finished formulations, HS3004) has increased from US $485.3 million in 2001 to US $1436 million in 2019 (Source: ITC Trade Map, 2020), and has been projected to increase further (BMI Research, 2019: 20). The monetary value of imports was almost six times that of exports in 2018 (BMI Research, 2019: 20). Most manufacturing companies interviewed reported doing very little exporting from South Africa. That which does take place both draws on significant levels of imported inputs (e.g., Fridge 2000, 48; Maloney & Segal, 2007: 17) and is small in value, targeting the regional market. Of finished drugs sold in the domestic market in South Africa, the imported share increased from 15% in 1990 to 30% in 2000 (Fridge, 2000: 168). By 2006, imports were estimated at 41% of the value of the local market, with 59% formulated locally (Maloney & Segal, 2007: 28).

**Figure 2:**
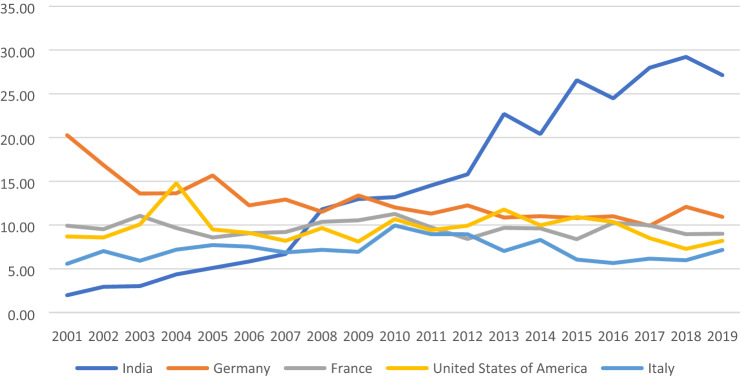
Share of South Africa’s pharmaceutical formulation imports, 2001–2019. Source: Compiled from analysis of HS 3004 data from ITC TradeMap. The selected countries are the top 5 source countries for South African imports of these products in 2019.

With European and American MNEs largely concentrating production at centers of excellence elsewhere, competition from India has been widely cited as the major competitive challenge for that formulation manufacturing which remains in South Africa. India is the number one source of imports of pharmaceutical formulations into South Africa. Figure 2 demonstrates India’s growing share of South Africa’s imports of pharmaceutical formulations. The then Director of Pharmaceuticals at the DTI acknowledged that India is not the biggest contributor to the trade deficit when compared to aggregate imports from Europe and the USA, but argued it is the biggest competitor to domestic manufacturing:

“The major contributor to the imports burden are imports of innovator and branded products from Europe and the USA. There is little competition with the domestic industry in this market segment. India, which tops the list of importers, is the supplier of generic medicines and competes directly with the domestic industry” (Kudlinski, 2013: 271).

The DTI has claimed that the South African pharmaceutical industry “can compete against imports from any country except India” (DTI, 2011: 4). The introduction of mandatory generic substitution in 2003 was influential in the subsequent shift in composition of the South African industry toward generics (Gray, Suleman, & Pharasi, 2017), a segment of the industry where India has considerable expertise (Athreye, Kale, & Ramani, 2009; Chaudhuri, 2005; Horner, 2014; Joseph, 2016). One South African policymaker commented on India’s pharmaceuticals, “they present an image as missionaries, the ‘pharmacy of the developing world’. It's fantastic for ARVs, but at the same time they’re undermining the capacity of our generic industry” (Interview, Pretoria, July 24, 2014). Another South African policymaker even went as far as to say that, in pharmaceuticals, the “Indian industry has killed off South African industry. There’s no doubt” (Interview, Cape Town, February 27, 2019).

Many companies supplying generic pharmaceuticals rely almost exclusively on imports of finished formulations, especially from India, with little or no manufacturing and only registration and marketing presence in South Africa. As relative exceptions, both Cipla and Ranbaxy (now part of Sun Pharmaceuticals) have manufacturing presence in South Africa, having acquired and upgraded plants from other companies exiting manufacturing. Although the former only fully acquired Cipla-Medpro in 2013, it had a distribution relationship with Medpro since the 1990s, and took over a manufacturing plant of the British-owned MNE Reckitt and Benckiser in Durban in 2005. Ranbaxy began selling products in South Africa in 1997 and acquired Be-Tabs, a South African firm established at Rooodeport in 1974, in 2007. However, they are part of a very small minority of Indian-owned firms which have established manufacturing in South Africa. For strategic reasons, as well as local regulatory requirements, many Indian firms formed partnerships with local entities for marketing, registration, and distribution. One interviewee explained how difficulties in market access meant Indian firms “joined hands” with local firms (Interview, Johannesburg, October 29, 2014). Another suggested that while Indian companies specialized in manufacturing, “local expertise and local knowledge” was crucial for sales and marketing (Interview, Sandton, July 23, 2014). Finally, a number of interviewees explained the benefits of local partnerships included addressing concerns around Indian medicines, especially regarding the quality (e.g., Interview, Centurion, July 17, 2014; also Interview, Midrand, October 30, 2014; Interview, Cape Town, July 30, 2014).

A wide range of interviewees emphasized the importance of sourcing from, and having relationships with, firms in India, especially for those that do have local manufacturing presence in South Africa. One interviewee explained that 70% of their sales value is imported from India and the rest is manufactured locally, although indicated that his company:

“would actually like to move what we do locally to India. I’d like someone else to worry about the manufacture. I’ve got my headache around here trying to chase people. So manufacture isn’t a key business for me” (Interview, Midrand, July 18, 2014).

Another interviewee from a South African firm with Indian ownership suggested “every single company in SA has some or more products from India or some or more content from India” (Interview, Sandton, October 30, 2014). Yet another explained that “I don’t think there’s a single company in South Africa that’s not involved with India…. They’re here to stay” (Interview, Pretoria, November 3, 2014; also Interview, Cape Town, July 30, 2014). The CEO of a South African firm with ties to India simply said: “If you’re in generics, you’ve got to have Indian partners” (Interview, Midrand, July 22, 2014). Although Aspen and Adcock Ingram have invested directly in India (Gelb, 2014), for the most part South African firms have sourced from and formed ties with companies in India to leverage Indian production. The managing director of a company which contract manufactures from India referred to a visit to India in the late 1990s or early 2000s:

“When I walked through those Indian factories, the level of innovation, the technology was 5–10 years ahead of SA. And that’s when it hit me, that it would take us a huge amount of capital investment to get our factories to that level to compete on the same level. So I thought it’s not gonna happen.…. So I mean at that point, I thought I don’t think we can beat them. If you can’t beat them, join them” (Interview, Johannesburg, July 15, 2014).

Interviewees identified a number of constraints for pharmaceutical firms manufacturing in South Africa. For example, the Country Head (for South Africa) of another Indian firm suggested that “They’d rather make it there and send it here. that’s the issue. …They err on the side of importing because it’s more cost-effective” (Interview, Midrand, July 16, 2014). Input costs such as water, electricity and labor were pointed to as higher in South Africa (Interview, Sandton, July 23, 2014). Moreover, some interviewees noted that the South African market appears relatively small for some Indian companies, not offering significant volumes for greater front-end investment (Interview, Midrand, July 18, 2014; Interview, Centurion, July 16, 2014). The CEO of an industry association group said succinctly, in relation to India, “they’ve got scale and here we don’t” (Interview, Midrand, July 16, 2014). With much larger volumes, a related advantage is the full range of related industries in India, especially presence in the API stage of the value chain. For example, the Managing Director of an Indian-owned firm in South Africa observed that in India “equipment, APIs, packing materials, everything is locally available. For SA today, we don’t have that. We don’t have any major bulk drug companies” (Interview, Johannesburg, October 29, 2014; also Interview, Pretoria, November 3, 2014). While some representatives of South African-owned firms suggested importing Indian firms benefited from greater state facilitation compared to local companies, through tax incentives (Aspen, 2010: 7), representatives of Indian companies did not acknowledge any such benefits (Interview, Midrand, July 24, 2014; NAPM, 2010: 17).

The trends outlined in this section – of both the decline of manufacturing in South Africa by MNEs and increasing import reliance – are both products, to a considerable degree, of the liberalization of the South African economy and concentration of production for GVCs elsewhere. The next section explores how the South African state has sought to promote better development outcomes in this context.

## STATE RESPONSES: FROM FACILITATOR TO PRODUCER AND BUYER

This section demonstrates the struggles of the South African state in promoting better development outcomes regarding pharmaceuticals. The facilitator role, primarily driven by the DTI, and its limitations are first discussed. In light of the struggle to promote greater manufacturing using the facilitator role, this section then proceeds to examine the state’s engagement with the producer role as well as the influential buyer role. The regulator role, the fourth in the four-fold typology of state roles in GVCs (Horner, 2017), is very important in pharmaceuticals, including as noted above in terms of generics availability and in quality approvals. However, it has not been adopted for industrial policy purposes in this case, so is not featured in its own sub-section here.

Before proceeding, it is important to note that these state initiatives are shaped by dual motivations. In reviewing South Africa’s 1996 National Drug Policy, “an unresolved tension between the NDP’s economic and health objectives” was observed (Gray et al., 2017: 54). Moreover, the DTI has noted the “absence of [an] integrated plan between relevant Government Departments re local production of pharmaceuticals” (2017: 16). Different, and sometimes conflicting, priorities are thus manifest in the discussion below.

### Facilitating Local Manufacturing

Drawing on various claims of the benefits of local pharmaceutical production, local manufacturing interests have argued for state incentives to facilitate such activity. Aspen, the largest South African-owned pharmaceutical firm and that which has consistently had the largest market share in the domestic market over the last two decades (BMI Research, 2019), has been credited with pushing the state to provide such assistance (Sprague & Woolman, 2008: 12). Pharmaceuticals Made in South Africa (Pharmisa), with Aspen playsing an influential role, was also formed to push for such support. Pharmisa has pointed to benefits of local production, including supply certainty, skills, new technologies and export potential (Focus Reports, 2012: 36).

One of the first major initiatives by the DTI to support local pharmaceutical manufacturing was the Strategic Industrial Projects (SIP) scheme in the early-mid 2000s. The key projects supported are illustrated in Table 5 below. As well as the MNE Roche, the SIP scheme supported South Africa’s two largest pharmaceutical firms, Aspen Pharmacare and Adcock Ingram. The former’s investment in manufacturing was notable given their original plan to sell off the manufacturing business after acquiring South African Druggists in 1999 (Sprague & Woolman, 2008: 5).

**Table 5:** DTI support for investment in pharmaceutical manufacturing under the Strategic Industrial Project scheme

Company	Year	Project	Capital investment (*R* mn)	Size of tax allowance (*R* mn)
Roche	2003	Spartan, Johannesburg Fansidar plant	110	79.5
Aspen	2003	Port Elizabeth oral solid dosage facility	360	110.7
Aspen	2005–2008	Port Elizabeth Steriles project for multi-drug resistance TB	370	164.5
Adcock Ingram	2008	Wadeville and Clayville plants	915, including 505 on ARV capacity	458

Source: Adapted from DTI (2011: 21–23)

Pharmaceuticals has been a priority sector under the more sectorally targeted industrial policy South Africa has pursued since 2007 when the Industrial Policy Acton Plan (IPAP) was initially launched (TIPS, 2016) in an attempt to diversify the South African economy and to promote structural transformation. Along with chemicals and plastic fabrication, pharmaceuticals was identified as one of four lead sectors in the original IPAP, and it has continued to receive special attention in subsequent iterations. Recognizing that import penetration was a threat (DTI, 2007: 9), the targeting of the sector made it eligible for preferential access to government investment incentives and procurement for boosting manufacturing. In the first 10 years following the launch of the initial IPAP in 2007, plastics, pharmaceuticals and chemicals received the third largest support by sector from the Industrial Development Corporation, after primary minerals beneficiation and green industries for renewable energy (DTI, 2018: 3).

A variety of subsequent initiatives have been attempted by the DTI to support pharmaceutical manufacturing. The Manufacturing Competitiveness Enhancement Programme was launched in 2012 focused on existing operations, to facilitate improving technology and equipment through a range of tax and cash allowances. Plastics and pharmaceuticals has received the most investment with the DTI’s Black Industrialists Scheme, with grants of R567 million for assistance following the launch in November 2015 (DTI, 2018: 42). Plans have been initiated to develop the Dube Trade Port into a pharmaceutical cluster (DTI, 2016), through infrastructure and services provision as well as tax incentives. Cipla subsequently announced it would expand some of its local manufacturing there (DTI, 2016; Kahn, 2020).

The DTI’s role as a facilitator has encountered various difficulties in its operation, with some firms which received incentives experiencing performance difficulties. For example, the DTI (2014: 94) admitted that “the pharmaceutical industry revival programme, assisted by DTI incentives (tax allowance of R813 million awarded under the SIP programme between 2003 and 2008) brought mixed results”. The government department said that Adcock Ingram hadn’t recovered the R2.5 billion investment in manufacturing plant upgrading between 2008 and 2012 due to loss of state tenders and private market share (DTI, 2014: 94). A policymaker interviewed explained that:

“in the case of the SIP, [it] didn’t happen. It was a number of spectacular failures. …. Roche got this money and three years later shut down the factory” (Interview, Pretoria, July 24, 2014).

The same interviewee cited examples of other companies which struggled and ultimately conceded that: “Pharma have been part of the Industrial Policy Action Plan right from the onset. But not much is happening” (Interview, Pretoria, July 24, 2014).

Critics have questioned whether the South African state could, and should, play a facilitator role vis-à-vis local manufacturing. Paul Anley, former CEO of Pharma Dynamics (which Indian-owned Lupin acquired a 60% stake of in 2008, and 100% ownership in 2015), argued that government incentives were insufficient to supersede the market incentives for importation of finished products:

“the benefits of economies of scale of global manufacturing far outweigh any potential incentives for local production. The inefficiencies of smaller production runs to satisfy local demand are greater than the incentives provided by government” (PharmaExec, 2016: 11).

The CEO of a South African company complained about the lack of incentives vis-à-vis the level of competition from India (Interview, Midrand, July 22, 2014). Others have queried whether the state should play a facilitator role. Arguments that doing so will improve the trade balance (NAPM, 2010: 14) or will lead to significant job creation (Maloney & Segal, 2007: 13) have both been questioned. Moreover, arguments that local production of formulation will increase supply security have been countered with the point that “security is in API production” (NAPM, 2010; Focus Reports, 2012: 16), and thus greater presence in the formulation stage would make little difference in this regard. Finally, the benefits for access to medicines have also been questioned, particularly if locally produced products are more expensive (Tomlinson & Low, 2012; Interview, Cape Town, July 24, 2014). Thus, the “made in SA debate” (Focus Reports, 2012: 15) has been contested and, as this section has demonstrated, the state has struggled in its facilitator role.

### The State as Producer

In an attempt to encourage the local industry by addressing the lack of manufacturing presence in the API stage of the value chain, the South African state has attempted to play a producer role through setting up a state-owned company. Long discussed (e.g., Fridge, 2000: 204), the idea took particular momentum following the African National Congress’ 2007 resolution to set up a state-owned company to manufacture pharmaceuticals (Kahn, 2018). Cross-department feasibility studies were conducted and identified APIs for ARVs, such as efavirenz, emtricitabine, tenofovir and lamivudine, as having potential due to the volumes consumed in South Africa (Maloney & Segal, 2007: 63). In 2012, plans for an API plant, 50% owned by the state and with funding from the DoH and the Industrial Development Cooperation, were announced under the name of project “Ketlaphela” (meaning ‘I will live or survive’ in Sesotho). The DTI (2012) declared ““Ketlaphela will reduce the country’s dependence on imported drugs and will provide security of supply of priority drugs, stable pricing with less sensitivity to exchange”. The DTI (2014: 97) itself acknowledged that “the rationale of Project Ketlaphela is more strategic than economic”, with the main stated aim being “security of supply of ARVs”.

The plan to established state-owned API production has received criticism from some representatives of the generics industry as well as access to medicines campaigners. The National Association of Pharmaceutical Manufacturers (NAPM) (2010) questioned the feasibility of an API plant due to the lack of scale, citing as an example Wellcome’s opening, and subsequent closing, of a trimethoprim API plant in South Africa in the 1980s. Members of the renowned Treatment Action Campaign cautioned against the potential for higher prices and reduced access to medicines through public procurement favoring Ketlaphela and its suppliers. They also questioned the job creation potential of the project, and suggested that import of medicines under patent was a bigger contributor to the trade deficit (with Germany, the US, France and the UK also major sources of imports to South Africa) than importation of APIs (Tomlinson & Low, 2012).

Despite being mooted for many years, the producer role for the state has struggled to get off the ground. An initial plan involved the Swiss company Lonza in collaboration with Pelchem and the Industrial Development Corporation and was due to come on stream in 2016 (DTI, 2012). The partner company, Lonza, withdrew in 2013 and further expressions of interest for commercial partners were sought. Since the initial plans were made, the imported cost of APIs had fallen (Interview, Pretoria, July 24, 2014). Another call for expressions of interest for business partners with Ketlaphela was issued in 2018 (Kahn, 2018) and the project has continued to attract attention in light of the COVID-19 pandemic (Tomlinson, 2020).

### State as Buyer

The role of the state as a buyer, through public procurement, has become a central focus point in efforts to promote greater local production of pharmaceuticals in South Africa. Public sector demand is very significant, as noted earlier being worth 31% of the value, and the majority of the volume, in the industry in 2017 (Helen Suzman Foundation, 2018: 3). The volumes in the market are significant for potentially encouraging local manufacturing, especially given the relevance of economies of scale to pharmaceuticals production (e.g., Interview, Sandton, July 24, 2014). Moreover, the public market is associated with having greater policy scope vis-à-vis the private market, with one interviewee acknowledging that “it is not easy to control the private market at all” (Interview, policymaker, Cape Town, February 27, 2019). As demonstrated in Table 6, this market is dominated by certain categories of drugs, notably ARVs for which South Africa has the highest incidence (over 7 million people) and biggest treatment programme in the world (Low & MacDonnell, 2019). The centralized buying power of the South African government has achieved considerable price decreases over time by negotiating aggressively with suppliers (Suleman & Gray 2017: 295; Wouters, Sandberg, Pillay, & Kanavos, 2019: 369).

**Table 6:** Top nine category expenditures of medicines supplied to the public sector, 2017

	Number of active ingredients	Public expenditure (*R* million)	Total procurement (%)
Anti-retrovirals	17	5453	36.6
Vaccines	10	2436	15.6
Anti-bacterials	32	991	6.7
TB drugs	10	441	3.0
Diabetes	5	402	2.7
Epilepsy	9	376	2.5
Anti-hemorrhagics	5	294	2.0
Perfusion solutions	11	257	1.7
Analgesics	4	227	1.5

Adapted from: Helen Suzman Foundation (2018: 11). In total, the nine medicines above accounted for 72.3% of the value of medicines supplied to the public sector in 2017

Public procurement has become a key focus of industrial policy in pharmaceuticals, as well as in other sectors, in South Africa. The country did not sign up to the 1994 WTO Agreement on Government Procurement (GPA) and so “is free to use government procurement as an industrial policy tool” (Kudlinski, 2013: 274), as well as to overcome prior discrimination (Bolton, 2006). The Preferential Procurement Policy Framework was designed to promote contracting with those previously discriminated against as well as to deliver the aims of the Reconstruction and Development Programme (Fridge, 2000: 65), while the Broad-based Black Economic Empowerment Act 53 of 2003 includes a code of good practice for preferential procurement. The New Growth Path (NGP), launched in 2010, and Industrial Policy Action Plan (IPAP) identified public procurement as “one of the key industrial levers” (DTI, 2014: 42).

Pharmaceuticals public procurement has been reformed substantially post-apartheid and has been largely driven by a health interest rather than the industrial interest of the DTI and local manufacturers. The previously fragmented public procurement system in pharmaceuticals was centralized post-apartheid and since 2010 has been solely managed by the Department of Health (Pharasi et al., 2010). The original 1996 National Drug Policy recognized a number of aims for public procurement: “Preference will be given to national manufacturers. Notwithstanding this preference, procurement will aim at securing the lowest available prices for products of defined specifications” (Government of South Africa, 1996: 14). A maximum 15% price preference was identified for “the national pharmaceutical manufacturing industry”, citing World Bank recommendations (Government of South Africa, 1996: 15). The DTI has argued in favor of procurement from local formulation manufacturers, pointing to benefits such as flexibility and security of supply, responsiveness and better quality control (DTI, 2011: 4 and 17). Nevertheless, they have lamented that in reality procurement has been too much focused on lowest cost, wherever the supply comes from (DTI, 2014: 42; also Kudlinski, 2013; Interview, Midrand, 30th October 2014). In explaining the priority of the health interest, Aaron Motsoaledi, Minister for Health 2009–2019, explained in relation to ARVs that: “there is no choice. We must purchase ARVs at the lowest possible cost from whatever source that can guarantee us the lowest prices, whether it’s inside the country or outside the country” (Keet, 2010).

Preferential procurement to local manufacturers has been attempted through both allowing price premiums and designation of specific products for local manufacturers. Tenders are issued every 2–3 years for each category of medicine. Although the majority of points (90) in the tender scoring system have been allocated to the lowest-cost supplier, up to ten points have been awarded as preference to domestic manufacturers (DTI, 2011: 9). Amendments to the Preferential Procurement Policy Framework Policy Act in 2011, however, promoted designation of procurement for local manufacturers in certain sectors (DTI, 2011: 9; Kudlinski, 2013: 274). The first designated pharmaceutical tender (Oral Solid Dosage, OSD, code HP-09) was in April 2012 (DTI, 2014: 98). Price premiums for local manufacturers are since on an “ad hoc basis” (Wouters et al., 2019: 364). For example, for HP13 2015 (the ARV tender), there was a 90:10 rule (as allowed per PPFA) with preference for local manufacturers, who would be negotiated with if within 10% of the highest points scorer (DTI, 2017: 23).

Considerable frustration and contestation have emerged over the implementation of preferential procurement, however. A number of representatives of local manufacturers complained of inconsistency in relation to the preferences for local production (e.g., Interview, Midrand, October 30, 2014). Uncertainty over government tenders has been cited by the DTI as one challenge for manufacturing (DTI, 2011: 4), as the 2-year tender system “does not allow for long-term investment planning” (DTI, 2011: 17). Policymakers also experienced frustration with the response of local manufacturing firms to the tenders. Two interviewees recalled how, even when a significant number of products were designated for local manufacturing, local firms did not always bid for the tenders (Interview, Johannesburg, July 15, 2014; Interview, Pretoria, July 24, 2014).

Imports from India, especially, have continued to play a prominent role in the public market as well, with differing reactions by industry and health interests. The extent of imported content in pharmaceutical tenders is thought to be high, with Aspen (2010: 10) suggesting 53%, while an informed policymaker suggested 65% of such procurement comes from India (personal correspondence, July 3, 2014; see also some estimates at DTI, 2011: 11). For HP13 2015 (the ARV tender), local manufacturers were awarded 62% of the tender, or R8.7 bn (DTI, 2017: 23). Estimates of the extent of local content are difficult to assess, however, given the extent of imported ingredients and the fact that locally owned companies can source some of their formulations from abroad. Nevertheless, it is widely recognized that “the major competition to domestic manufacturers in government tenders comes from imports from India” (DTI, 2011: 9). While the Director of Pharmaceuticals at the DTI referred to “crowding out” in national tenders by Indian imports (Kudlinski, 2013: 259), the then South African Minister of Health has complemented the contributions of the Indian industry to South Africa stating that “we regard India as the pharmacy of the developing world” (Krishnan and Gahlot, 2015).

Nevertheless, the significance of the buyer role of the state is pointed to by the fact that many companies which are major contributors to South Africa’s public procurement have some local manufacturing. In value terms, the largest suppliers to the public sector in 2017 (along with the main drug or therapeutic area they are in the list for) were Biovac Institute (vaccines supplier), Aspen Pharmacare (HIV), Mylan (HIV), Sonke Pharmaceuticals (HIV), Cipla Medpro (HIV), Sanofi Aventis (TB, diabetes and epilepsy) and AbbVie (HIV) (Helen Suzman Foundation, 2018: 3). With the exception of AbbVie, which is unclear, all have some local manufacturing in South Africa (company websites, accessed March 4, 2020). Sanofi describes itself “as one of a few multinational companies with a local manufacturing plant” (Sanofi website, accessed March 7, 2020). In terms of the major suppliers by number of products to the public sector, along with Aspen Pharmacare, Cipla Medpro and Sanofi from the above list, Fresenius Kabi and Adcock Ingram are also in the top five and have local manufacturing (Helen Suzman Foundation, 2018: 3). As well as benefiting from the state’s facilitator initiatives, Aspen’s manufacturing expansion has been attributed to demand for ARVs (public and private) (Sprague and Woolman, 2008: 6). One claim suggested that some foreign firms had bought local factories, with limited value-added activities, to improve their bargaining with government (including on tenders) (Maloney & Segal, 2007: 89). Investments by foreign firms in ARV manufacturing in South Africa which have been linked to facilitating participation in the public tender business include Ranbaxy and Cipla in the mid-2000s (DTI, 2011: 22), Sandoz in 2012 (Palmer, 2012), Mylan’s plant acquisition from Ascendis Health in 2019 (BMI Research, 2019: 4) and Laurus’ acquisition of Aspen-subsidiary Phekolong Pharmaceuticals in 2019 (Njobeni, 2019).

Although the buyer role of the state of the state is very prominent in relation to South Africa’s pharmaceutical industry, and is subject to considerable tension between health and industrial interests, it has become a key focus of industrial policy. Despite variations in incentives, the South African state’s buyer power appears to have played a role in encouraging some formulation manufacturing within the country.

## CONCLUSION

The case of South Africa’s pharmaceutical industry is one where a deindustrialization trend has been observed over the last two and a half decades in a context of trade liberalization and the consequent emergence of an import-oriented engagement with GVCs. A consolidation of production by lead firms, including European and American MNEs as well as Indian generic producers, in GVCs has posed considerable challenges for pharmaceutical manufacturing in South Africa. Lead firms have primarily engaged with South Africa as an end market to sell already finished products into, rather than as a location for establishing manufacturing operations. The pharmaceutical production which is present in the country is almost exclusively focused on formulations production, with almost no presence in the active pharmaceuticals ingredients (API) stage of the value chain, which accentuates the challenge for expanding manufacturing. Taking account of the context of competing with pharmaceuticals produced via GVCs reveals the scale of the task involved and provides for a more nuanced account of the possibilities of local manufacturing (or not).

In response, the South African state has made various uses of the facilitator, producer and buyer roles, motivated by both health and industry interests. The industrial policymaking efforts have been targeted less at export orientation and mostly at the more immediate target of preventing loss, and potentially reclaiming presence, of locally produced pharmaceuticals in the domestic market. The facilitator role has been deployed to support a number of manufacturing facilities, although has struggled to transform the prospects for domestic manufacturing. For more than a decade, the producer role has been explored to move upstream into the API stage of the value chain, yet has still not been fully realized. The buyer role, although largely driven by health interests and constrained by tensions between the consumer and industrial interest, is one which appears to have had more influence in encouraging major suppliers to public procurement to retain or establish local manufacturing.

By highlighting the development implications of import-oriented engagement with GVCs, and associated state policymaking, this article builds on and goes beyond the valuable prior insights in development studies, economic geography, economic sociology and international business on export-oriented economic development and state policy-making in relation to GVCs. As demonstrated here, local manufacturing in places outside major centers of production for GVCs faces a significant challenge from the threat of crowding-out by imported products. This is influential in curtailing the maintenance or development of local territorial assets, which could have potentially been leveraged in an export-oriented context in the future. Moreover, the article has conceptually advanced understandings of the role of the state vis-à-vis GVCs, beyond the facilitator role assumed in export-oriented participation in GVCs to also explore the producer and buyer role. This case has demonstrated the latter, which has received less previous attention in relation to GVCs and the state, to be a very active industrial policy tool due to the size of the market and policy scope it is associated with.

The article has wider relevance for understanding of, and efforts to address, the challenge of contemporary (de-) industrialization. The contemporary liberalized trade context and existence of GVCs places constraints on industrialization and state development strategies, even those which ‘just’ seeks to serve domestic markets – arguably the dominant and most relevant strategy in many manufacturing contexts outside major production locations in Asia. Under-analyzed to date, more research is needed in other countries and sectors to examine the prospects for development under import-oriented forms of engagement with GVCs.
